# Effect of IGF-1 C472T, GH C2141G, and GHR T914A polymorphisms on growth performance and feed efficiency in young Kazakh white-headed cattle

**DOI:** 10.14202/vetworld.2023.1584-1592

**Published:** 2023-08-10

**Authors:** Nikolay P. Gerasimov, K. M. Dzhulamanov, S. V. Lebedev, V. I. Kolpakov

**Affiliations:** Federal Research Centre of Biological Systems and Agrotechnologies RAS, Orenburg, Russian Federation

**Keywords:** feed efficiency, growth hormone gene, growth hormone receptor gene, insulin-like growth factor-1 gene, Kazakh white-headed breed, polymorphism

## Abstract

**Background and Aim:**

Improving the feed efficiency of beef cattle is necessary to increase the profitability of meat production. Implementing marker-assisted selection breeding systems can improve the genetic potential of beef cattle for increased productivity. This research aimed to study the effects of insulin-like growth factor (IGF)-1 C472T, growth hormone (GH) C2141G, and GH receptor (GHR) T914A polymorphisms on growth performance and feed efficiency in young Kazakh white-headed cattle.

**Materials and Methods:**

Young Kazakh white-headed cattle (n = 50) were grouped after weaning according to sex (28 bulls and 22 heifers) and they were genotyped according to the IGF-1 C472T, GH C2141G, and GHR T914A polymorphisms. The test period was conducted from 8 to 15 months of age. The experimental animals were evaluated for live weight (LW) at the beginning and end of the test period. They were also assessed for average daily gain, hip height, metabolic mid-weight (MMWT), actual dry matter intake (DMI), and residual feed intake (RFI).

**Results:**

Significant differences in MMWT were found between the bulls with the IGF-1^TT^ and IGF-1^CT^ genotypes, which was a 2.2 kg increase in heterozygous cattle (p < 0.05). Heterozygous IGF-1^CT^ bulls differed with a higher DMI of 0.087 kg/day (p < 0.05) compared to IGF-1^TT^ homozygotes. Carriers of the IGF-1^TT^ genotype had the greatest feed efficiency at 0.068 kg/day (p < 0.05). Heifers with the GH^CC^ genotype differed in their maximum DMI with an increase of 1.17%–1.57% (p < 0.05) relative to the other genotypes. The G allele in the GH C2141G polymorphism was associated with better (p < 0.05) feed efficiency in the Kazakh white-headed breed. The minimum DMI and RFI in GHR T914A heterozygous heifers were significantly inferior (p < 0.05) to the other genotypes.

**Conclusion:**

Association studies of the IGF-1 C472T, GH C2141G, and GHR T914A polymorphisms indicate a relationship between growth, development, and feed efficiency with the genetic characteristics of young Kazakh white-headed cattle. A significant (p < 0.05) dominant effect was found in the IGF-1 gene in bulls and in the GHR gene in heifers, which should be considered when breeding with heterogeneous parental pairs. The negative effect of the allele substitution in the IGF-1 C472T polymorphism was observed in the LW of heifers (−3.25 kg) at the age of 8 months and bulls (−6.05 kg) at 15 months. The substitution in the GH C2141G polymorphism was associated with a significant reduction in DMI by 0.036 kg (p < 0.05) and an increase in feed efficiency by 0.023 kg (p < 0.05) during the rearing of heifers. These results can improve the production efficiency of mature herds of Kazakh white-headed cattle.

## Introduction

The main purpose of selection in beef cattle is the evaluation and subsequent concentrated use of animals with the best breeding and economic value for herd reproduction. The genetic progress in a beef herd is largely determined by the method of selection for the replacement animals, which is systemic in nature and includes several successive stages from weaning to introduction into the mature herd [1]. The period of growth and development is a relatively small part of the animal’s life; however, the features of its formation, especially at the pubertal stage of maturation, have an irreversible impact on their further breeding and economic use [2]. The Kazakh white-headed cattle breed was created by crossing and combining the best qualities of native Kazakh and Kalmyk cattle and their crosses with Hereford sires. The breed was created for rearing in the dry steppe and semi-desert zones to effectively use the large areas of natural pastures. Particular importance was attached to the fattening qualities, the ability to quickly restore body condition after heavy wintering and maintaining it for a long period of time, and to travel long distances. The outstanding qualities of Kazakh white-headed cattle include high adaptation and unpretentiousness to various feeding and rearing conditions, and good reproductive traits for the taste and nutritional properties of the beef meat have established their widespread distribution in Russia and Kazakhstan [3]. For a long time, the Kazakh white-headed breed was improved by linear breeding, which involved strict selection, primarily in the males of the herd, for the control of the genealogical structure and the initial evaluation of young animals was performed according to its pedigree [4]. As a result, the current state of the genetic resources in Kazakh white-headed breed tends to be in reduced diversity, which ultimately results in a decrease in phenotype variability. Thus, Soloshenko *et al*. [5] evaluated the effect of the low level of genotype variability in the main breeding traits, which reflected the high degree of consolidation in Kazakh white-headed cattle. The implementation of marker-assisted selection (MAS) in breeding systems can improve the genetic potential of beef cattle for increased productivity [6]. For example, the selection and concentrated reproduction of carriers with the “preferred” genotypes can increase the annual selection effect by 15%–30% compared to traditional breeding methods [7]. The lifetime evaluation of beef cattle is based on the measurements of live weight (LW), average daily gain (ADG), constitution, and body conformation [8, 9]. Numerous studies show that these traits have relatively high heritability [10, 11]. This provides evidence for genetic factors that determine economic and beneficial traits in cattle [12]. Genetic markers such as single-nucleotide polymorphisms (SNPs) are the most common form of DNA variation in farm animals. They are numerous, usually biallelic, and are relatively easy to determine using proven methods [13]. These properties explain the high potential of SNPs used in beef cattle breeding to improve the economic efficiency of the industry. In addition, the molecular genetic markers may have a pleiotropic effect, which is expressed through the simultaneous determination of several independent economic traits [14]. Multiple genes control the physiological regulation of quantitative traits in beef cattle [15]. Genes of the somatotropic axis (e.g., growth hormone [GH], GH receptor [GHR], and insulin-like growth factor [IGF]) are of particular importance to improve the genetic potential of beef cattle, as they are associated with the growth, development, and differentiation of tissues, the intensity of metabolic processes, and animal lactation [16, 17]. It should be considered that the listed biological processes are determined polygenically, that is, by several genes. Therefore, it is advisable to consider polymorphisms of several genes for a single physiological characteristic during selection using genetic markers. The bovine GH gene is located on chromosome 19, is approximately 1793 base pairs in length, and consists of five exons and four introns [18]. The GH g.2141C > G (GH L127V) polymorphism is a non-synonymous amino acid substitution from leucine to valine (Leu > Val) and occurs at codon 127 of the gene. The GHR gene is located on chromosome 20 in cattle and encodes for a transmembrane GHR belonging to a large superfamily of cytokine and hematopoietic GHRs [19, 20]. Genetic analysis revealed ten polymorphic regions in the bovine GHR gene [21]. The substitution of the T→A nucleotide in exon 8 (GHR T914A) causes an amino acid sequence change from phenylalanine to tyrosine (Phe > Tyr) at codon 279 (F279Y). Another important factor in the somatotropic axis is the IGF-1 or somatomedin gene, which plays a key role in various physiological and metabolic processes within the body. The IGF-1 gene was mapped on chromosome 5 of cattle and consists of six exons [22, 23]. A polymorphism in the 5’-flanking region of exon 1 is IGF-1/SnaBI (IGF-1 C472T), which is caused by a T→C nucleotide substitution and is recognized by the SnaBI restrictase [12, 24].

Considering the vast range of phenotypic data of beef cattle affected by polymorphisms in the somatotropic axis genes, we hypothesized that genetic features would influence the animal’s nutrient and energy requirements related to body weight gain. This is indirectly evidenced by the presence of a sufficiently high degree of heritability of the residual feed intake (RFI) (h^2^ = 0.33 range of 0.07–0.62) in cattle [25]. Improving the feed efficiency of beef cattle through breeding is necessary to increase the profitability of meat production [26–28].

This research aimed to study effect of the IGF-1 C472T, GH C2141G, and GHR T914A polymorphisms on growth performance and feed efficiency in young Kazakh white-headed cattle.

## Materials and Methods

### Ethical approval

All animal studies were performed in accordance with the instructions and recommendations of the Russian Regulations of 1987 (Order No. 755 on 12.08.1977 the USSR Ministry of Health) and “The Guide for Care and Use of Laboratory Animals (National Academy Press Washington, D.C. 1996).” Every possible effort was made during the research to minimize animals’ distress and use fewer samples.

### Study period and location

The study was conducted from October 2021 to April 2022 at the test station of the Plemzavod “Krasny Oktyabr” Agricultural Production Cooperative (Pallasovsky District, Volgograd Region, Russia).

### Sample collection and analysis

Young Kazakh white-headed cattle were genotyped according to IGF-1 C472T, GH C2141G, and GHR T914A polymorphisms. DNA was isolated from whole blood using the “DIAtom™ DNAPrep” kit (IsoGeneLab, Moscow, Russia). The quality of the isolated DNA was evaluated using horizontal electrophoresis SE-2 (Helicon, Russia) on a 1.5% agarose gel “Agarose, Biotechnology grade” (Helicon). Genotyping was performed by polymerase chain reaction (PCR)-Restriction Fragment Length Polymorphism on a “Tertsik” programable thermal cycler (DNA technology, Russia) to determine the polymorphisms in the IGF-1, GH, and GHR genes using primers designed by the Scientific and Production Company “Litekh” Ltd. (Russia). A quantitative PCR was performed using Bio-Rad CFX 96 (Bio-Rad, Hercules, CA, USA). The following components were combined in a 0.2 mL reaction tube: 5 μL ready-made PCR mixture qPCRmix-HS (Evrogen, Russia), 1 μL of 5 μmol forward primer, 1 μL of 5 μmol reverse primer, 2 μL of DNA template, and deionized water up to a total volume of 25 μL. Polymerase chain reaction conditions are described in Table-1 [12, 29, 30].

**Table-1:** Primer sequence, PCR conditions, and restriction enzymes used in genotyping.

SNP	Amplicon, bp	Primer	PCR conditions	Restriction enzymes	Reference
IGF-1 C472T	249	F: 5’- attacaaagctgcctgcccc-3’ R: 5’- accttacccgtatgaaaggaatatacgt-3’	Initial heating – at+95°C for 3 min; 35 cycles: denaturation – at+95°C for 30 s; annealing – at+64°C for 30 s; synthesis – at+72°C for 30 s; primer extension – at+72°C for 10 min	SnaBI	[12]
GH C2141G	428	F: 5’- gctgctcctgagccttcg-3’ R: 5’- gcggcggcacttcatgaccct-3’	Initial heating – at+95°C for 5 min; 35 cycles: denaturation – at+94°C for 45 s; annealing – at+65°C for 45 s; synthesis – at+72°C for 45 s; primer extension – at+72°C for 7 min	AluI	[29]
GHR T814A	182	F: 5’- atatgtagcagtgacaatat-3’ R: 5’- acgtttcactgggttgatga-3’	Initial heating – at+95°C for 5 min; 35 cycles: denaturation – at+95°C for 30 s; annealing – at+60°C for 60 s; synthesis – at+72°C for 30 s; primer extension – at+72°C for 10 min	SspI	[30]

SNP=Single-nucleotide polymorphism, PCR=Polymerase chain reaction, IGF-1=Insulin-like growth factor-1, GH=Growth hormone, GHR=Growth hormone receptor

The resulting products were separated by horizontal electrophoresis in a 1× Tris-borate buffer at 80 V in a 2.5% agarose gel for 30 min. and stained with ethidium bromide. The gel was visualized using a “UVT-1” transilluminator and photographed using the “VITran v.1.0” (“Biokom”, Russia) system. Fragment length was determined according to the “GenePakR DNA Ladder M 50” (IsoGeneLab) molecular-weight size marker.

### Experimental design

Young Kazakh white-headed animals (n = 50) were grouped after weaning according to sex (28 bulls and 22 heifers). The average weaning age was 205 days. Animals were provided with a preparatory stage (average 38 days) from weaning until the age of 8 months to acclimatize to the feeding and housing conditions. The test period was conducted from 8 to 15 months of age in a typical station in a light-type and free-stall barn with straw as bedding. Animals were fed in a feed yard with free access to water.

Feeding rations for the bulls consisted of mixed grass hay, haylage, barley corn, and sunflower meal, and for the heifers smooth brome hay, mixed grass hay, haylage, and barley corn (Table-2). Feed was provided *ad libitum* and contained 11.5%–12.5% crude protein and 8.45–9.54 MJ metabolizable energy per 1 kg of dry matter.

**Table-2:** Feed and nutrient intake by young animals (test period 213 days).

Indicator	Group	

	Heifers	Bulls
Smooth brome hay, kg	1413.7	-
Mixed grass hay, kg	372.4	902.4
Haylage, kg	639.0	1604.0
Barley corn, kg	256.5	354.0
Sunflower meal, kg	-	354.0
Feed contains		
Dry matter, kg	1691.1	1882.0
Metabolizable energy, MJ	14289.7	17960.1
Crude protein, kg	212.0	214.7
Digestible protein, kg	120.0	158.3
Metabolizable energy on a dry matter basis, MJ kg^-1^	8.45	9.54

The experimental animals were evaluated for LW at the beginning (Initial LW) and end (Final LW) of the test period. They were also assessed for ADG, hip height (HH), metabolic mid-weight (MMWT), actual dry matter intake (DMI), and RFI. The weight growth of the young animals was monitored monthly during the test period (213 days). The ADG for each animal was calculated as the linear regression coefficient of the LW for the duration of the test period. The MMWT was calculated as the midpoint of the LW during the test period to the power of 0.73 (MMWT^0.73^). HH was measured using a Lydtin measuring stick at 15 months of age. Individual feed intake was determined daily according to the difference between the provided and residue amount of feed. Chemical analysis of the feed mixture was performed monthly to determine the dry matter content by drying samples (500 g) at 100°C in an oven. Organic matter was determined by ashing the dried sample at 550°C [31]. The average daily DMI was calculated by dividing the actual DMI consumed over the test period by the duration of the test period. Data on the weight, linear growth, and feed intake according to the groups are presented in Table-3.

**Table-3:** Overall trait means and standard error in experimental young Kazakh White-headed animals.

Indicator	Group	

	Bulls	Heifers
n	28	22
Initial live weight, kg	234.6 ± 3.58	213.2 ± 2.63
Final live weight, kg	428.7 ± 5.14	348.8 ± 3.51
Average daily gain, g	911.0 ± 28.93	636.6 ± 18.40
Hip height, cm	123.4 ± 0.68	120.1 ± 0.64
Metabolic mid- weight, kg^0.73^	69.2 ± 0.49	61.3 ± 0.38
Dry matter intake, kg/day	8.876 ± 0.0159	7.923 ± 0.0199

Expected DMI was determined by solution of a multiple regression considering actual DMI, ADG, and MMWT using the generalized linear models procedure according to the following model:


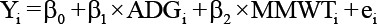


where Y_i_ – expected DMI, kg; β_0_ – regression intercept; β_1_ – partial regression coefficient of DMI on ADG; β_2_ – partial regression coefficient of DMI on MMWT; ADG_i_ – ADG from 8 to 15 months, kg; MMWT_i_, kg^0.73^; e_i_ – residual error in DMI.

### Statistical analysis

The effects of the genotypes on the traits studied were analyzed using the least-squares method according to the general linear model procedure of Statistics 10.0 software (“Stat Soft Inc.,” USA).

Model used:


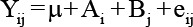


where, Y_ij_ – represents the studied traits, μ – is the overall mean, A_i_ – is the fixed effect of the IGF-1, GH, GHR genotype (1, 2, 3), B_j_ – is the fixed effect of the sex (1, 2), and e_ij_ – is random error.

The effects of the sire and dam on the performance of the offspring were not incorporated into the statistical model because a single sire was used for the artificial insemination of cows of the same age, from the same herd. The significance of the intergenotype differences was assessed using the posteriori Fisher’s criterion (F-test). p ≤ 0.05 was considered statistically significant.

The additive and dominant effects and the allele substitution effect were calculated using models according to those previously described by Falconer and Mackay [32].

## Results

The IGF-1 C472T polymorphism determined the variability in weight growth of young Kazakh white-headed cattle (Table-4). Heifers with the IGF-1^CC^ genotype were significantly (p < 0.05) superior in their initial LW compared to heterozygous and homozygous carriers of the T allele by 14.5 kg (6.91%). However, there were no significant differences between the initial LW of the bulls with the various genotypes. However, bulls with the IGF-1^TT^ variant were significantly inferior in their Final LW by 26.2 kg (5.96%; p < 0.05) compared to the heterozygous carriers at the end of the test period. This trend was confirmed in the heifers, but was less pronounced.

**Table-4:** Growth performance and feed efficiency in young Kazakh White-headed cattle depending on IGF-1 C472T polymorphism (M ± m).

Indicator	IGF-1 C472T genotype					

	CC	CT	TT	CC	CT	TT
	
	Bulls			Heifers		
n	6	12	10	5	10	7
Initial LW, kg	233.0 ± 8.07	236.1 ± 5.98	233.9 ± 5.76	224.4 ± 1.96ab	209.9 ± 4.19a	209.9 ± 4.15b
Final LW, kg	433.0 ± 10.77	439.4 ± 5.71a	413.2 ± 9.59a	350.4 ± 5.78	350.9 ± 4.07	344.6 ± 8.92
ADG, g	939.2 ± 80.69	954.7 ± 36.98	841.7 ± 44.88	591.6 ± 22.81	662.1 ± 27.65	632.4 ± 37.95
Hip height, cm	123.8 ± 1.49	124.6 ± 0.88	121.8 ± 1.23	119.8 ± 1.59	120.6 ± 0.81	119.6 ± 1.32
MMWT, kg^0.73^	69.4 ± 0.62	70.1 ± 0.65a	67.9 ± 0.97a	62.3 ± 0.56	61.2 ± 0.46	60.7 ± 0.90
DMI, kg/day	8.888 ± 0.0354	8.912 ± 0.0202a	8.825 ± 0.0248a	7.940 ± 0.0164	7.906 ± 0.0316	7.921 ± 0.0445
RFI, kg/day	0.009 ± 0.0163	0.033 ± 0.0194a	−0.035 ± 0.0285a	0.041 ± 0.0148	−0.010 ± 0.0246	−0.015 ± 0.0310

^a^Values in a row with the same indices differ with significance p *<* 0.05, ^b^p < 0.05. IGF-1=Insulin-like growth factor-1, CC=Genotype CC, CT=Genotype CT, TT=Genotype TT, n=Number of animals, LW=Live weight, ADG=Average daily gain, MMWT=Metabolic mid-weight, DMI=Dry matter intake, RFI=Residual feed intake

A noted improvement in the ADG of heterozygous individuals for the bulls was 15.5–113.0 g (1.65%–13.43%; p > 0.05) and for heifers was 29.7–70.5 g (4.70%–11.92%; p > 0.05) as determined by analyzing the ADG for the test period (8–15 months). In addition, the heterozygous animals had better linear measurements than the homozygous cattle, which were demonstrated by the greater HH of 0.8–2.8 cm (0.65%–2.30%; p > 0.05) in bulls.

Significant differences in the MMWT were found between the bulls with the IGF-1^TT^ and IGF-1^CT^ genotypes, which was a 2.2 kg increase in the heterozygous cattle (3.24%; p < 0.05). The relatively high mass of the heterozygous bulls was associated with a higher DMI of 0.087 kg/day (0.99%; p < 0.05) compared to the TT homozygotes. However, carriers of the IGF-1^TT^ genotype had the greatest feed efficiency at 0.068 kg/day (p < 0.05). Differences in DMI and RFI were not significant (p > 0.05) between the heifer genotypes, although the heterozygous and homozygous carriers of T allele demonstrated an advantage over the CC homozygotes.

No significant effect (p > 0.05) of the GH C2141G polymorphism was found on the weight and linear growth in young Kazakh white-headed cattle (Table-5). There was a tendency for the superiority of G allele carriers in terms of LW, ADG, and HH. Heifers with the GH^CC^ genotype differed in the maximum DMI during the test period with an increase of 0.092–0.123 kg (1.17%–1.57%; p < 0.05) relative to the other genotypes. This trend was confirmed in the group of bulls, but the effect was less pronounced.

**Table-5:** Growth performance and feed efficiency in young Kazakh White-headed cattle depending on GH C2141G polymorphism (M ± m).

Indicator	GH C2141G genotype					

	CC	CG	GG	CC	CG	GG
	
	Bulls			Heifers		
n	13	11	4	10	9	3
Initial LW, kg	235.1 ± 5.01	232.7 ± 6.95	238.5 ± 5.63	214.5 ± 3.89	210.6 ± 4.81	216.7 ± 2.96
Final LW, kg	425.8 ± 7.44	429.2 ± 9.60	436.7 ± 8.28	342.8 ± 5.32	352.7 ± 5.10	357.0 ± 9.64
ADG, g	895.4 ± 47.30	922.3 ± 48.51	930.7 ± 39.62	602.4 ± 27.28	667.3 ± 29.20	658.7 ± 32.84
Hip height, cm	122.9 ± 0.98	123.5 ± 1.26	124.7 ± 1.03	118.9 ± 1.05	121.0 ± 0.87	121.3 ± 1.20
MMWT, kg^0.73^	69.0 ± 0.59	69.1 ± 1.01	70.1 ± 0.86	60.9 ± 0.58	61.4 ± 0.61	62.2 ± 0.98
DMI, kg/day	8.892 ± 0.0207	8.866 ± 0.0305	8.852 ± 0.0342	7.973 ± 0.0182ab	7.881 ± 0.0312a	7.850 ± 0.0656b
RFI, kg/day	0.022 ± 0.0151	-0.010 ± 0.0301	-0.020 ± 0.0313	0.037 ± 0.0112a	-0.028 ± 0.0289a	-0.038 ± 0.0473

^a^Values in a row with the same indices differ with significance p *<* 0.05, ^b^-p < 0.05. GH=Growth hormone, CC=Genotype CC, CG=Genotype CG, GG=Genotype GG, n=Number of animals, LW=Live weight, ADG=Average daily gain, MMWT=Metabolic mid-weight, DMI=Dry matter intake, RFI=Residual feed intake

The G allele in the GH C2141G polymorphism was associated with better feed efficiency in the Kazakh white-headed breed. A pronounced advantage of G allele carriers was recorded among the heifers, which was 0.065–0.075 kg (p < 0.05). The differences between the bulls in the RFI were 0.032–0.042 kg (p > 0.05) in favor of the G allele carriers.

The young Kazakh white-headed cattle with the AA genotype for the GHR T914A polymorphism were characterized by the maximum weight and linear growth; however, the intergroup differences did not reach a significant (p > 0.05) level (Table-6). Heterozygous individuals had the highest ADG over the test period. The minimum DMI was found in the heterozygous heifers, which was significantly reduced by 0.130 kg/day (1.63%; p < 0.05) compared to those with the TT genotype.

**Table-6:** Growth performance and feed efficiency in young Kazakh White-headed cattle depending on GHR T914A polymorphism (M ± m).

Indicator	GHR T914A genotype					

	TT	TA	AA	TT	TA	AA
	
	Bulls			Heifers		
n	12	9	7	6	10	6
Initial LW, kg	236.7 ± 5.62	230.0 ± 6.04	237.0 ± 7.90	210.7 ± 6.00	212.8 ± 3.38	216.3 ± 5.73
Final LW, kg	421.9 ± 8.58	432.6 ± 8.78	435.3 ± 9.39	337.3 ± 6.94	352.8 ± 5.42	353.5 ± 4.11
ADG, g	869.2 ± 38.81	951.0 ± 52.42	931.1 ± 68.7	594.7 ± 49.63	657.4 ± 20.52	644.0 ± 31.35
Hip height, cm	122.6 ± 1.11	123.8 ± 1.10	124.4 ± 1.39	118.7 ± 1.26	120.3 ± 1.11	121.2 ± 0.60
MMWT, kg^0.73^	68.8 ± 0.91	69.1 ± 0.77	69.9 ± 0.71	60.2 ± 0.61	61.6 ± 0.63	61.9 ± 0.59
DMI, kg/day	8.869 ± 0.0206	8.869 ± 0.0351	8.897 ± 0.0312	7.995 ± 0.0269a	7.865 ± 0.0273a	7.932 ± 0.0307
RFI, kg/day	0.006 ± 0.0203	−0.015 ± 0.0333	0.023 ± 0.0195	0.036 ± 0.0186a	−0.041 ± 0.0215ab	0.032 ± 0.0300b

^a^Values in a row with the same indices differ with significance p *<* 0.05, ^b^p < 0.05. GHR=Growth hormone receptor, TT=Genotype TT, TA=Genotype TA, AA=Genotype AA, n=Number of animals, LW=Live weight, ADG=Average daily gain, MMWT=Metabolic mid-weight, DMI=Dry matter intake, RFI=Residual feed intake

Young animals with homozygous GHR T914A genotypes consumed dry matter feed less efficiently. A significant superiority of the heterozygous genotype for the GHR T914A polymorphism was found in heifers, which amounted to an increase of 0.073–0.077 kg (p < 0.05). A similar genotype superiority distribution was recorded among the bulls, but the advantage was 0.021–0.038 kg (p > 0.05).

Table-7 shows the allele substitution effects of the somatotropic axis genes for traits with significant (p < 0.05) differences between the various genotypes in Kazakh white-headed cattle. A negative effect of the C allele for T substitution at the IGF-1 gene locus on weight growth was observed in bulls, which was expressed as a reduced LW at 15 months by 6.05 kg (p = 0.023) and MMWT by 0.5 kg^0.73^ (p = 0.050) compared to the other genotypes. Moreover, a significant (p < 0.05) decrease in the mass of the bulls was noted with the homozygous T allele, which indicates the dominance of the substitution effect. A strong dominant effect of the T allele over C allele at the IGF-1 gene locus was established through the consumption and efficiency of feed intake. This allele substitution was accompanied by decreased DMI by 0.020 kg (p = 0.013) and improved feed efficiency by 0.014 kg (p = 0.037).

**Table-7:** Allele substitution effect estimates and estimates of additive and dominance effects of SNP studied showing significant associations with traits in young Kazakh White-headed cattle.

Trait	SNP	Allele substitution effect	Additive effect	Dominant effect	Overall P-value
Bulls					
Final LW	IGF-1 C472T	−6.05	−9.9	16.3*	0.023
MMWT	IGF-1 C472T	−0.5	−0.75	1.45*	0.050
DMI	IGF-1 C472T	−0.020	−0.0315	0.0555*	0.013
RFI	IGF-1 C472T	−0.014	−0.022	0.045*	0.037
Heifers					
Initial LW	IGF-1 C472T	−3.25	−7.25*	−7.25	0.029
DMI	GH C2141G	−0.036	−0.0615*	−0.0305	0.024
RFI	GH C2141G	−0.023	−0.0375*	−0.0275	0.047
DMI	GHR T914A	−0.016	−0.0315	−0.0985**	0.005
RFI	GHR T914A	−0.003	−0.002	−0.075*	0.031

* Effect is significant with p *<* 0.05,

** p < 0.01. SNP=Single-nucleotide polymorphism, IGF-1=Insulin-like growth factor-1, GH=Growth hormone, GHR=Growth hormone receptor, LW=Live weight, MMWT=Metabolic mid-weight, DMI=Dry matter intake, RFI=Residual feed intake

The negative effect of the substitution of the C allele for T at the IGF-1 gene locus on weight growth was confirmed in the heifers, which was expressed as a decrease in LW by 3.25 kg at 8 months (p = 0.029). The substitution of the C allele for G allele in the GH C2141G polymorphism was associated with an improvement in the fattening performance of Kazakh white-headed heifers. There was a significant reduction in DMI by 0.036 kg (p = 0.024) and an increase in feed utilization during rearing of 0.023 kg (p = 0.047). The fattening traits increased with the homozygous presence of the G allele, which indicated a significant additive effect of the nucleotide substitution. A similar positive effect of allele substitution was noted at the GHR gene locus, but with a significant (p < 0.05) dominant effect of A allele over T allele.

## Discussion

This study considers the growth and fattening performance of Kazakh white-headed cattle with different genotypes of the GH, GHR, and IGF-1 genes. The products of these genes are regulatory proteins that constitute the hormonal series of the somatotropic axis. The biological function of proteins of the somatotropic axis is associated with the regulation of metabolism, growth and differentiation of tissues and organs, lactation, and other physiological processes. The involvement of endocrine factors in the development of animals necessitates the use of polymorphisms in their encoded genes for breeding strategies to improve the fattening qualities of beef cattle. Thus, weight and linear growth, feed consumption, and rearing efficiency were studied depending on the IGF-1 C472T, GH C2141G, and GHR T914A polymorphisms in young Kazakh white-headed cattle. These polymorphisms are located on bovine chromosomes 5, 19, and 20. Nkrumah *et al*. [33] noted the presence of the quantitative trait loci for *Bos taurus* autosome (BTA) 5 (ADG, feed efficiency), BTA 19 (ADG), and BTA 20 (DMI). Abo-Ismail *et al*. [34] found a strong association of the sites on BTA 19 with the fattening qualities in crossbred beef cattle. Karisa *et al*. [35] identified the quantitative trait loci for body weight, feed efficiency, and energy metabolism on BTA 20 that were associated with polymorphisms in the GHR gene.

This study aimed to determine the potential of using polymorphisms in the IGF-1, GH, and GHR genes for MAS in Kazakh white-headed cattle for fattening efficiency. This will accelerate the genetic improvement of the breed by increasing the accuracy of selection and reducing the interval between the generations of evaluated animals [36].

The use of the IGF-1 C472T polymorphism in the MAS of beef cattle is explained by its effect on the phenotypic variability in weight growth and meat quality [37–39]. Thus, the T allele showed a dominant effect over the C allele in terms of pre-slaughter LW and post-weaning ADG in beef cattle [12, 40]. This study found a significant dominant effect in the LW at 15 months of age and MMWT in bulls, which explained 19.5% and 14.6% of the phenotypic trait variability, respectively. Bulls carrying the TT genotype differed with the lowest (p < 0.05) DMI. The effect of the BB genotype (TT in this study) on the minimum feed intake of young animals was confirmed in the study by Siadkowska *et al*. [37] on Holstein-Friesian cattle. A negative effect of the allele substitution on weight growth at weaning was found in Kazakh white-headed heifers. As a result, large CC animals consumed more feed and were less efficient in control rearing than the alternative genotypes. This was partly confirmed in the bulls by the minimum RFI (−0.035 kg) of homozygous TT individuals. Ardicli *et al*. [39] and Siadkowska *et al*. [37] also established the relative profitability of rearing animals with the TT genotype.

Selionova and Plakhtyukova [41] found the effect of amino acid substitution Leu → Val at codon 127 of the GH gene on the variability of the LW and ADG when studying the differences in weight growth of Kazakh white-headed bulls. In this case, young animals with the VV genotype (GG in our case) had a significant (p < 0.05–0.01) advantage in body weight at weaning and 1 year of age, as well as in the growth rate during the control rearing period. The results of this associative analysis are consistent with the data obtained in this study on evaluating the effectiveness of rearing Kazakh white-headed cattle. Thus, substituting C → G alleles explained 12.3%–13.8% of the phenotypic variability of weight growth in heifers, but among the bulls, this parameter was lower and varied by 5.0%–6.0%. In this substitution, G allele carriers outperformed the CC homozygous cattle in terms of LW at 15 months of age and ADG for the period of control rearing. Lee *et al*. [42] found a significant (p < 0.05) superiority of GG homozygotes in the growth rate of Hanwoo cattle, which is consistent with our results. However, Sedykh *et al*. [16] noted the increased mass of carriers of the GH^LL^ genotype in Hereford and Limousine bulls, which significantly outperformed the other genotypes by 4.95% and 4.18% (p < 0.01) at 20 months of age, respectively. Thus, the relationship between GH C2141G polymorphism and weight growth in beef cattle appears to be breed and population-specific. The relative mass of the body in G allele carriers did not require high feed costs; therefore, CG and GG individuals consumed the least amount of dry matter during the control rearing period. A significant (p < 0.05) additive effect of the nucleotide substitution was observed in the heifers, which determined 31.0% of the variability of DMI and 22.7% of RFI. The rank of the genotype distribution in bulls reiterated the trend of GG > CG > CC according to the GH C2141G polymorphism, as established for the feed efficiency in heifers. Despite that the GH C2141G polymorphism is a widespread candidate marker to improve meat and milk production, information regarding its association with fattening efficiency is limited. Therefore, further research is required on the relationship between the GH gene and feed efficiency in cattle, which could provide a significant contribution to beef breeding programs.

In the MAS of beef cattle a significant role is assigned to polymorphisms in the GHR gene. Nametov *et al*. [43] identified the desirable YY (AA in this study) genotype of the GHR F279Y polymorphism in Kazakh white-headed bulls, which outperformed the FF (TT in this study) homozygous cattle in the LW at 18 months of age by 7.12% and at 20 months of age by 7.37%. The difference in weight growth was minimal between the homozygous and heterozygous Y allele carriers. In turn, the differences in LW between the FF and YY genotypes were less pronounced by 1.4%–1.7% in favor of the GHR^YY^ genotype in Aberdeen Angus bulls [44]. Carriers of the AA genotype (YY) significantly (p < 0.05) outperformed the TT genotype (FF) cattle by 7% in the average daily weight gain of heifers [19]. This research confirms the previously identified tendency for increased weight growth in Y allele carriers. Therefore, it can be considered as “desirable” for the selection of the Kazakh white-headed cattle breed. In addition, studies in beef cattle have shown a significant (p = 0.026) association of polymorphisms in the GHR gene with the RFI [35]. According to previous analyses on feed efficiency indicators, the dominance (−0.1353 kg/day) of heterozygous individuals was demonstrated compared to the homozygous genotype cattle. Sherman *et al*. [15] found a significant dominant effect of the A allele over the G allele on LW, ADG, feed consumption, and efficiency in beef cattle with the polymorphism at intron 4 of the GHR gene. However, Abo-Ismail *et al*. [34] noted the association of the minor allele of the GHR gene with a decrease in RFI. In this study, a significant (p < 0.05) dominant effect was also found with the superiority of the heterozygous genotype in terms of feed consumption and fattening efficiency in heifers. In this case, the effect of allele substitution provided 35.6% and 28.3% of the variability for the DMI and RFI traits, respectively.

## Conclusion

Association studies of the IGF-1 C472T, GH C2141G, and GHR T914A polymorphisms indicate a relationship between growth, development, and feed efficiency with the genetic characteristics of young Kazakh white-headed cattle. A significant (p < 0.05) dominant effect was found in the IGF-1 gene of bulls and the GHR gene of heifers, which should be considered in the mating of heterogeneous parental pairs. The negative effect of the allele substitution in the IGF-1 C472T polymorphism was observed in the LW of heifers (−3.25 kg) at the age of 8 months and bulls (−6.05 kg) at 15 months. The substitution of C allele to G allele in the GH C2141G polymorphism is associated with a significant reduction in the DMI by 0.036 kg (p < 0.05) and an increase in feed efficiency by 0.023 kg (p < 0.05) while rearing heifers.

## Authors’ Contributions

NPG, KMD, SVL, and VIK: Contributed equally to the experimentation. NPG and KMD: Wrote and edited the manuscript. SVL and KMD: Equally designed and conducted the experiment. NPG and VIK: Studied scientific literature about the topic. All authors have read, reviewed, and approved the final manuscript.
